# Ethical conflicts in patient care situations of community pharmacists: a cross-sectional online survey

**DOI:** 10.1007/s11096-024-01797-9

**Published:** 2024-09-06

**Authors:** Kathrin Wernecke, Stephan Nadolny, Jan Schildmann, Susanne Schiek, Thilo Bertsche

**Affiliations:** 1 Institute for History and Ethics of Medicine, Interdisciplinary Center for Health Sciences, Martin Luther University Halle-Wittenberg, Magdeburger Strasse 8, 06112 Halle (Saale), Germany; 2 Institute for Educational and Health-Care Research in the Health Sector, Hochschule Bielefeld -University of Applied Sciences and Arts, Interaktion 1, 33619 Bielefeld, Germany; 3 Clinical Pharmacy Department, Institute of Pharmacy, Medical Faculty, Leipzig University, Bruederstrasse 32, 04103 Leipzig, Germany; 4 Drug Safety Center, Leipzig University and Leipzig University Hospital, Bruederstraße 32, 04103 Leipzig, Germany

**Keywords:** Community pharmacy, Cross-sectional study, Ethics, Ethical issues, Pharmacy ethics, Survey

## Abstract

**Background:**

Community pharmacy practice is rarely considered in ethical research, although various ethical conflicts are known for this setting. Data on the actual frequency and perceived burden of ethical conflicts occurring in the community pharmacy setting are required.

**Aim:**

The survey aimed at investigating the frequency and perceived burden of ethical conflicts, reasons for the perceived burden and influences on decision-making in ethical conflicts in German community pharmacists.

**Method:**

An online survey was conducted among community pharmacists. It contained 15 ethical conflicts in which the ethically required action conflicts with another principle (e.g. law). Basing on these conflicting principles, 12 considerations relevant for decision-making were defined (e.g. solidarity principle). Participants were asked to rate the ethical conflicts in terms of frequency and perceived burden and to rate the influence on decision-making for the considerations. Results were analysed descriptively.

**Results:**

Five hundred and thirty-five questionnaires were evaluated. The participant’s median age was 39 (min–max: 20–78) years, 378 (71%) were female. Seven of the 15 predefined ethical conflicts were rated as occurring predominantly at least once a week. “Generic drug is not most suitable” was rated as the most frequent. Three ethical conflicts were rated mainly with a (very) strong burden. “Concerns for an unborn child” was rated as the most burdensome. Three of the 12 predefined decision-making considerations: pharmaceutical knowledge, legal requirements and personal values were rated primarily as having a very strong influence on decision-making.

**Conclusion:**

Pharmacists in community pharmacies are frequently affected by burdensome ethical conflicts in patient care situations.

**Supplementary Information:**

The online version contains supplementary material available at 10.1007/s11096-024-01797-9.

## Impact statements

- To reduce the perceived burden and develop possible solutions for ethical conflicts, the most obvious starting point is to discuss them with colleagues or in a team meeting in the pharmacy.
- Offering more ethical content in pharmacy education and further training is desired by pharmacists and has the potential to prevent them from negative consequences such as distress.
- Both, ethical discussion and ethical education and training, should be promoted through exchange with other professional groups (e.g. medical ethicists).
- Further research is also needed in this area in order to better address the specific ethical aspects of community pharmacy.

## Introduction

Community pharmacy practices faces various ethical problems. Ethical problems can be broadly defined as conflicts regarding moral values or norms relevant to patient care either because of an ethical deficit, misconduct or due to uncertainty about the appropriate course of action [1]. Ethical conflict particularly arises in a community pharmacy if the ethically required action conflicts with another principle of work [2]. Typical principles involved in conflicts in this setting are legal and organizational regulations, economic considerations, time pressure, and the collaboration with and/or demarcation from other professional groups [3–5]. The latter is the case in situations with strongly divergent opinions of other health care professionals. Thereby conflict solution can be complicated by the spatially and substantively separated work of community pharmacists. Additionally, self-care with self-medication can raise specific ethical conflicts, for example, when over-the-counter (OTC) analgesics are misused by the patient [2]. Care situations in which the pharmacist interacts directly with the patient have an especially high potential for ethical issues [6].

However, community pharmacy practice is rarely considered in ethical research [7]. There is a paucity of data to date on how often community pharmacists are confronted with ethical conflicts or how much they are burdened by them. The frequency and perceived burden are both important parameters regarding the impact of ethical conflicts [3, 8, 9]. A prolonged and frequent burden can lead to distress and, thus, to a reduced quality of work, job dissatisfaction or emotional illness [10, 11]. To reduce the potential distress, an ethical decision-making process is necessary to solve the conflicts satisfactorily [12]. The reason for the perceived burden of an ethical conflict is an important starting point. The pharmacist’s decision will be influenced by how onerous they perceive the violation of one aspect or another. Such data on the influence of different considerations are rare but important to understand ethical decision-making undertaken by community pharmacists.

### Aim

Little is known generally about all the named parameters of ethical conflicts in a community pharmacy: the frequency and perceived burden, reasons for the perceived burden and influences on the decision-making in an ethical conflict. The aim of this survey was, therefore, to explore these data among German community pharmacists.

### Ethics approval

The study protocol was approved by the Ethics Committee of the Medical Faculty of Leipzig University (439/20-ek; October 13, 2020). The online survey was anonymous and no personal data were collected.

## Method

Methods are presented following the Checklist for Reporting Results of Internet E-Surveys [13]. An expert panel of three clinical pharmacists and two medical ethicists was responsible for developing the questionnaire and conducting the entire study.

### Setting and participants

The survey was conducted among pharmacists working in community pharmacies in Germany. Pharmacists in an internship during their last year of education were also included. Other possible professions in German community pharmacies are pharmacy business assistants, pharmacy technicians and pharmacy engineers. They were deliberately not addressed since pharmacists supervise them, also in case of an ethical conflict.

### Development of the ethical conflicts

Ethical conflicts were defined as situations in which an important principle of work (e.g. legal requirements) conflicts with the action ethically required [2]. The questionnaire included 15 ethical conflicts and aimed at describing typical patient care situations in community pharmacy practices. Therefore, a preparatory survey was conducted in which ten pharmacists were asked in writing which ethical conflicts they could spontaneously think of with which pharmacists are confronted in their work especially in community pharmacies. The pharmacists were recruited within the working group that conducted the survey. All of them had work experience in community pharmacy. Additionally, we searched for common ethical conflicts identified in the literature [4, 14].

Ethical conflicts were included in the questionnaire if they were:

- mentioned in at least two sources (preliminary survey and one literature sources or both literature sources)
- applicable to community pharmacies in Germany (legal, socio-economic)
- representing a patient care situation

Situations referring to cognitive pharmaceutical services were deliberately not considered as implementation varies substantially in Germany and the survey aimed at measuring the basic status without these potentially more burdensome situations.

Conflicts were transferred into the German context where necessary. All conflicts were discussed in the expert panel. The final ethical conflicts are shown in Table 1.

**Table 1:** Ethical conflicts in detail [own translation – original descriptions are in German]

Short form	Ethical conflict
*Ethical conflicts referring to legal requirements*	
Urgent prescription with formal error	A patient has a prescription for an urgently needed drug. The prescription contains a relevant formal error that requires consultation with the physician. The prescribing physician is known but cannot be reached
Missing prescription for a needed drug	A patient requests an acute supply of a prescribed drug for which they does not currently have a prescription. They would like to submit this at a later date
Dispensing concerns despite physician consultation	Concerns about dispensing arise when you advise a patient on a prescription. You discuss the concerns with the physician. You cannot reach an agreement with the physician and the concerns remain
*Ethical conflicts referring to patient behaviour*	
Suspected abuse of an OTC drug	You suspect that a patient is abusing an OTC drug
Implausible off-label use of a prescribed drug	A patient is using a prescription drug off-label, but this off-label use does not seem plausible to you
*Ethical conflicts referring to the exchange/lack of information*	
Missing information on the patient	A customer wants to buy a drug for a third person for self-medication. When asked, they can hardly provide any information about the patient for whom the drug is intended
Patient has problems understanding important information	You have the impression that the patient did not understand the content of your advice on the drug and this could lead to incorrect administration^†^
Secrecy hinders exchange of information	Your duty of confidentiality prevents you from disclosing or obtaining information that would be important for the patient's care
*Ethical conflicts referring to cost reimbursement*	
Generic drug is not most suitable	The generic drug paid for by the health insurance is not the drug that is most appropriate for the patient from a pharmaceutical perspective^††^
Invoicing of pharmaceutical services	A patient would benefit from pharmaceutical services (e.g. medication review). For economic reasons, you would have to charge the patient for these^†††^
Health insurance requirements hinder adequate supply	Due to the requirements of the health insurance, the patient cannot be adequately provided with an urgently needed medical aid
Patient cannot pay	The patient is not or hardly able to pay for the needed drug or medical aid
*Ethical conflicts referring to the choice of a drug (alternative)*	
Unsuitable alternatives due to supply shortage	You cannot supply a patient with a drug due to a supply shortage. Several alternatives, less suitable for the patient, are available
Patient asks for an OTC drug not needed	A patient asks for an OTC drug. During the consultation, you conclude that the patient does not need the drug, but it would not harm them
Concerns for an unborn child	Drug therapy for a pregnant woman creates concerns for the unborn child

*OTC* Over-the-counter

^†^This refers to situations in which, despite adequate counselling by the pharmacist, doubts remain (e.g. in the case of cognitively impaired patients)

^††^There are discount contracts between health insurances and pharmaceutical industry in Germany. The generic drug, for which a discount contract exists, has to be dispensed preferably. Deviations from the contract have to be specifically justified by the dispensing pharmacist. In this case, there is a threat of a financial loss, as the health insurance might not accept the justification and, therefore, will not pay for the drug dispensed

^†††^The regular invoicing from pharmaceutical services via the health insurance was not possible at the time of the survey

### Development of the decision-making considerations

Different considerations are taken into account for decision-making depending on the principle of work conflicting with the ethical required action [2]. Twelve common decision-making considerations were identified in the literature and discussed in the expert panel [2–4, 15–17]. The final items were (in alphabetical order): commercial considerations, evidence from studies and guidelines, instructions from the pharmacy manager, instructions from the physician, legal requirements, my experience, my own moral values, patient’s wishes, the pharmacy’s personnel and time resources, pharmaceutical knowledge, the solidarity principle and religious beliefs.

### Questionnaire development and pretesting

The questionnaire was drafted and discussed again in the expert panel. Afterwards, a cognitive and a conventional pretest were conducted. The cognitive pretest was performed via a think-aloud and probing technique with four pharmacists with experience in a community pharmacy and one pharmacist in an internship. The questionnaire was subsequently pretested conventionally by 11 people (two pharmacists in internships, four employed pharmacists, one pharmacist branch manager, two pharmacy owners and two others). They were able to provide written comments on the questionnaire. Both pretests were performed with the digitalized form, therefore, the person pretesting also saw the layout and tested the handling. Adjustments were made based on the pretests. The wording was adjusted in all parts, especially to make the ethical conflicts more concrete. Further adjustments made after the pretests and the final questionnaire is presented in Supplement 1.

### Survey execution and recruitment

The survey was conducted by using a platform (soscisurvey.de) and presented on 15 pages (Supplement 1). If an answer was missing, the participant was asked if they wanted to complete the information or proceed without it (check box required). Participants could change their answers (back button available). All German Chambers of Pharmacists were contacted by sending a web link to the questionnaire via email. They were asked to forward the invitation to community pharmacies, community pharmacists and pharmacists in an internship working in a community pharmacy. A reminder for the survey was sent after two months. The survey started on 1 December 2020 and was closed on 15 April 2021. Participation in the survey was voluntary and no incentives were offered.

### Data analysis

Data were transferred to Microsoft® Excel® 365 and analysed descriptively. Participants with single missing responses (no complete missing pages) were included in the analysis; questionnaires with complete missing pages were excluded. Questionnaires were included if the participant took at least five minutes to complete it. Five minutes were considered sufficient due to the repetitive nature of the questions and the repetitive items. No statistical correction of answers (e.g. weighting) was performed. Data on the perceived burden in a conflict were excluded if the participant rated “never” experiences the conflict, as, in this case, the burden is not based on experience. We computed absolute and relative frequencies for all questions despite age and professional experience. Regarding those variables, we report the median with minimum and maximum values (min–max).

## Results

### Participants

The website was accessed 1265 times. A total of 823 participants started the questionnaire and 549 finished it. One questionnaire was excluded because the participant did not answer any question. A further 13 questionnaires were excluded because they were filled in by professional groups other than pharmacists or pharmacists in an internship. As the survey was only addressed at pharmacists and pharmacists in internship, the other professional groups were not assessed in detail. The remaining 535 questionnaires were evaluated. The socio-demographic data for the 535 participants evaluated are shown in Table 2.

**Table 2:** Socio-demographic data

Socio-demographic data (n_total_ = 535)	Number of participants/value (% of n_total_)
Age	
Median (min–max)	39 (20–78)
Gender [n (%)]	
Male	156 (29.2)
Female	378 (70.7)
Diverse	1 (0.2)
Employment contract [n (%)]	
Employed pharmacist	263 (49.2)
Branch managing pharmacist	55 (10.3)
Owner of pharmacy	148 (27.7)
Pharmacist in internship	69 (12.9)
Professional experience in years [median (min–max)]	10 (0–40)
Percentage of working time in patient care [n (%)]	
0–10	6 (1.1)
11–20	8 (1.5)
21–30	17 (3.2)
31–40	28 (5.2)
41–50	36 (6.7)
51–60	74 (13.8)
61–70	89 (16.6)
71–80	132 (24.7)
81–90	102 (19.1)
91–100	43 (8.0)
Federal state [n (%)]	
Bavaria	242 (45.2)
Baden-Wuerttemberg	147 (27.5)
Saxony	77 (14.4)
North Rhine-Westphalia	20 (3.7)
Other	50 (9.3)
Locality size [n (%)]	
Metropolis (> 100.000 citizens)	214 (40.0)
City (> 20.000 citizens)	113 (21.1)
Small city (> 5.000 citizens)	137 (25.6)
Rural community (< 5.000 citizens)	71 (13.3)

### Frequency of ethical conflicts

Table 3 shows the rated frequencies for the ethical conflicts queried. The conflict “generic drug is not most suitable” had the most ratings in the frequency category “at least once a day” with 152 participants (28.4%). It, therefore, is the most frequent conflict (defined as the highest rating in the category “at least once a day”). One hundred and eighty-six participants (34.8%) rated the conflict “secrecy hinders exchange of information” to occur never which represents the highest rating in this category. It therefore represents the least frequent conflict.

**Table 3:** Reported frequency of the ethical conflicts

	“Please decide spontaneously for each situation: How often do you experience the situation mentioned in your everyday professional life? Please select the option that is most likely to apply.” [n_total_ = 535]						
Ethical conflict (short)	“at least once a day” [n (% of n_total_)]	“at least once a week” [n (% of n_total_)]	“at least once a month” [n (% of n_total_)]	“at least once a quarter” [n (% of n_total_)]	“at least once a year” [n (% of n_total_)]	“Never” [n (% of n_total_)]	Not Specified [n (% of n_total_)]
*Ethical conflicts referring to legal requirements*							
Urgent prescription with formal error	98 (18.3)	221 (41.3)	145 (27.1)	51 (9.5)	14 (2.6)	6 (1.1)	0
Missing prescription for a needed drug	86 (16.1)	261 (48.8)	141 (26.4)	35 (6.5)	10 (1.9)	2 (0.4)	0
Dispensing concerns despite physician consultation	7 (1.3%)	31 (5.8)	127 (23.7)	133 (24.9)	151 (28.2)	85 (15.9)	1 (0.2)
*Ethical conflicts referring to patient behaviour*							
Suspected abuse of an OTC drug	70 (13.1)	200 (37.4)	138 (25.8)	84 (15.7)	38 (7.1)	5 (0.9)	0
Implausible off-label use of a prescribed drug	5 (0.9)	30 (5.6)	94 (17.6)	132 (24.7)	164 (30.7)	108 (20.2)	2 (0.4)
*Ethical conflicts referring to the exchange/lack of information*							
Missing information on the patient	89 (16.6)	194 (36.3)	148 (27.7)	62 (11.6)	33 (6.2)	9 (1.7)	0
Patient has problems understanding important information	44 (8.2)	136 (25.4)	137 (25.6)	116 (21.7)	72 (13.5)	29 (5.4)	1 (0.2)
Secrecy hinders exchange of information	13 (2.4)	35 (6.5)	68 (12.7)	109 (20.4)	122 (22.8)	186 (34.8)	2 (0.4)
*Ethical conflicts referring to cost reimbursement*							
Generic drug is not most suitable	152 (28.4)	200 (37.4)	112 (20.9)	54 (10.1)	10 (1.9)	7 (1.3)	0
Health insurance requirements hinder adequate supply	44 (8.2)	111 (20.7)	168 (31.4)	122 (22.8)	59 (11.0)	30 (5.6)	1 (0.2)
Patient cannot pay	26 (4.9)	94 (17.6)	147 (27.5)	138 (25.8)	95 (17.8)	34 (6.4)	1 (0.2)
Invoicing of pharmaceutical services	52 (9.7)	89 (16.6)	108 (20.2)	91 (17.0)	51 (9.5)	143 (26.7)	1 (0.2)
*Ethical conflicts referring to the choice of a drug (alternative)*							
Patient asks for an OTC drug not needed	69 (12.9)	216 (40.4)	164 (30.7)	62 (11.6)	13 (2.4)	11 (2.1)	0
Unsuitable alternatives due to supply shortage	124 (23.2)	172 (32.1)	151 (28.2)	59 (11.0)	23 (4.2)	6 (1.1)	0
Concerns for an unborn child	1 (0.2)	20 (3.7)	69 (12.9)	115 (21.5)	202 (37.8)	126 (23.6)	1 (0.2)

*OTC* Over-the-counter

### Perceived burden

**Fig. 1:**
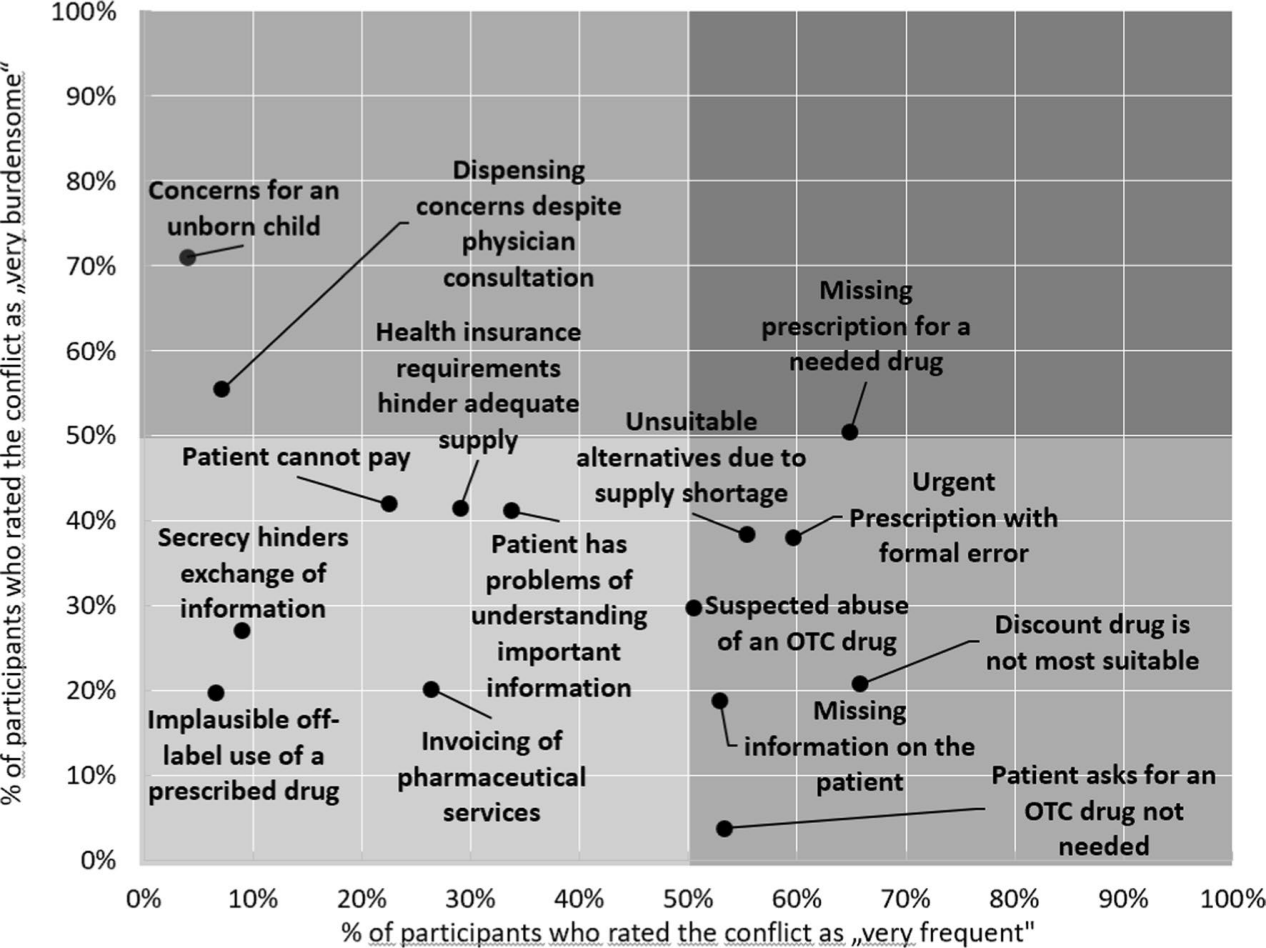
Comparison of the percentage of participants who rated the conflict as very frequent with the percentage of participants who rated the conflict as very burdensome (very frequent: “at least once a day” or “at least once a week” was rated; very burdensome: “very heavy burden” or “heavy burden “ was rated). *OTC* Over-the-counter

Table 4 shows the ratings for the perceived burden for the ethical conflicts and the reasons for the perceived burden. In the category with the highest burden (“very heavy burden”) most of the ratings were given to the conflict “concern for an unborn child” with 165 participants (30.4%). This conflict therefore can be considered as the most burdensome. Similarly, the conflict “patient asks for an OTC drug not needed” can be determined as the least burdensome with 103 (19.3%) ratings in the category “no burden”. The right part of Table 4 shows the results for the reasons for the perceived burden in participants who rated the conflict with a heavy or a very heavy burden. The fear for negative consequences for the patient was the mostly rated reason for the perceived burden in 13 out of the 15 conflicts. Figure 1 compares the percentage of participants who rated a conflict as very frequent (at least once a day or week) with those who rated them as very burdensome (heavy or very heavy burden).

**Table 4:** Reported perceived burden of the ethical conflicts [left] and reason for the perceived burden for conflicts rated with a (very) strong burden [right]

	“Please decide spontaneously for each situation: How much of a burden do you normally perceive in the situation mentioned? Please select the option that is most likely to apply.” [n_total_ = 535]								For participants who rated a (very) strong burden: “The situation is very burdensome because of the potential consequences for ….” (multiple categories possible)				
Ethical conflict (short)	“Very heavy burden” [n (% of n_total_)]	“Heavy burden” [n (% of n_total_)]	“Rather heavy burden” [n (% of n_total_)]	“Rather weak burden” [n (% of n_total_)]	“Weak burden” [n (% of n_total_)]	“No burden” [n (% of n_total_)]	Never experienced or not specified^†^ [n (% of n_total_)]	Total heavy or very heavy burden [n_heavy_]	“… the patient” [n (% of n_heavy_)]	“… the pharmacy” [n (% of n_heavy_)]	“… myself” [involved pharmacist, n (% of n_heavy_)]	Other involved parties^††^ [n (% of n_heavy_)]	No option applies [n (% of n_heavy_)]
*Ethical conflicts referring to legal requirements*													
Urgent prescription with formal error	69 (12.9)	132 (24.7)	146 (27.3)	99 (18.5)	63 (11.8)	20 (3.7)	6 (1.1)	201	166 (82.6)	117 (58.2)	103 (51.2)	“… the physician”: 5 (2.5)	2 (1.0)
Missing prescription for a needed drug	130 (24.3)	139 (26.0)	136 (25.4)	59 (11.0)	51 (9.5)	18 (3.4)	2 (0.4)	269	141 (52.4)	178 (66.2)	229 (85.1)	–	–
Dispensing concerns despite physician consultation	106 (19.8)	143 (26.7)	117 (21.9)	53 (9.9)	22 (4.1)	8 (1.5)	86 (16.1)	249	238 (95.6)	70 (28.1)	106 (42.6)	“… the physician”: 18 (7.2)	–
*Ethical conflicts referring to patient behaviour*													
Suspected abuse of an OTC drug	36 (6.7)	122 (22.8)	200 (37.4)	121 (22.6)	39 (7.3)	12 (2.2)	5 (0.9)	158	150 (94.9)	39 (24.7)	63 (39.9)	–	–
Implausible off-label use of a prescribed drug	12 (2.2)	72 (13.5)	132 (24.7)	142 (26.5)	47 (8.8)	10 (1.9)	188 (35.1)	84	81 (96.4)	12 (14.3)	26 (31.0)	“… the health insurance”: 1 (1.2)	–
*Ethical conflicts referring to the exchange/lack of information*													
Missing information on the patient	14 (2.6)	85 (15.9)	154 (28.8)	170 (31.8)	80 (15.0)	23 (4.3)	9 (1.7)	99	78 (78.8)	25 (25.3)	49 (49.5)	“… a third person”: 37 (37.4)	–
Patient has problems understanding important information	58 (10.8)	150 (28.0)	196 (36.6)	69 (12.9)	29 (5.4)	3 (0.6)	30 (5.6)	208	205 (98.6)	43 (20.7)	96 (46.2)	–	1 (0.5)
Secrecy hinders exchange of information	35 (6.5)	59 (11.0)	113 (21.1)	84 (15.7)	46 (8.6)	10 (1.9)	188 (35.1)	94	86 (91.5)	17 (18.1)	45 (47.9)	–	1 (1.1)
*Ethical conflicts referring to cost reimbursement*													
Generic drug is not most suitable	24 (4.5)	86 (16.1)	156 (29.2)	144 (26.9)	93 (17.4)	25 (4.7)	7 (1.3)	110	107 (97.3)	26 (23.6)	32 (29.1)	–	–
Health insurance requirements hinder adequate supply	83 (15.5)	126 (23.6)	171 (32.0)	81 (15.1)	34 (6.4)	9 (1.7)	31 (5.8)	209	205 (98.1)	43 (20.6)	36 (17.2)	“… the health insurance”: 1 (0.5)	–
Patient cannot pay	67 (12.5)	143 (26.7)	167 (31.2)	79 (14.8)	37 (6.9)	7 (1.3)	35 (6.5)	210	203 (96.7)	22 (10.5)	36 (17.1)	–	1 (0.5)
Invoicing of pharmaceutical services	20 (3.7)	59 (11.0)	116 (21.7)	115 (21.5)	56 (10.5)	25 (4.7)	144 (26.9)	79	70 (88.6)	23 (29.1)	19 (24.1)	–	–
*Ethical conflicts referring to the choice of a drug (alternative)*													
Patient asks for an OTC drug not needed	4 (0.7)	16 (3.0)	60 (11.2)	171 (32.0)	170 (31.8)	103 (19.3)	11 (2.1)	20	13 (65)	7 (35)	9 (45)	–	–
Unsuitable alternatives due to supply shortage	67 (12.5)	136 (25.4)	154 (28.8)	110 (20.6)	43 (8.0)	19 (3.6)	6 (1.1)	203	193 (95.1)	68 (33.5)	46 (22.7)	–	2 (1.0)
Concerns for an unborn child	165 (30.8)	124 (23.2)	73 (13.6)	29 (5.4)	13 (2.4)	3 (0.6)	128 (23.9)	289	247 (85.5)	65 (22.5)	132 (45.7)	“… a third person (child)”: 280 (96.9)	–

*OTC* Over-the-counter

^†^Data on the perceived burden in a situation were only considered if the participant stated in the previous question concerning the frequency that they experience the conflict at least once a year

^††^If another party (besides patient, pharmacy or myself) was involved in the situation, the potential negative consequences for them was also a selectable answer

Most frequent answer per ethical conflict is highlighted

### Decision-making considerations

Table 5 shows the rated influences on decision-making in a conflict for the 12 predefined items (left part of the table). The right part of the table shows the perceived burden in case of a violation of the queried item. The items were sorted according to the number of participants given the response "very strong influence" on decision-making. The order of the table can therefore be understood as a rating of the 12 items with pharmaceutical knowledge as the most influencing consideration and religious beliefs as the least influencing.

**Table 5:** Reported influence of different considerations on decision-making [left] and perceived burden in case of violation [right]

	“How strong is the influence of these considerations on your decision [in an ethical conflict situation] in everyday life? Please select the option that is most likely to apply.” [n_total_ = 535]							“How much of a burden do you feel when you ‘have to’ act against these considerations in everyday life? Please select the option that is most likely to apply.” [n_total_ = 535]						
	“Very strong influence” [n (% of n_total_)]	“Strong influence” [n (% of n_total_)]	“Rather strong influence” [n (% of n_total_)]	“Rather weak influence” [n (% of n_total_)]	“Weak influence” [n (% of n_total_)]	“No influence” [n (% of n_total_)]	Not specified [n (% of n_total_)]	“Very heavy burden” [n (% of n_total_)]	“Heavy burden” [n (% of n_total_)]	“Rather heavy burden” [n (% of n_total_)]	“Rather weak burden” [n (% of n_total_)]	“Weak burden” [n (% of n_total_)]	“No burden” [n (% of n_total_)]	Not specified [n (% of n_total_)]
Pharmaceutical knowledge	241 (45.0)	225 (42.1)	56 (10.5)	10 (1.9)	1 (0.2)	0	2 (0.4)	187 (35.0)	199 (37.2)	108 (20.2)	32 (6.0)	4 (0.7)	3 (0.6)	2 (0.4)
Legal requirements	230 (43.0)	170 (31.8)	112 (20.9)	18 (3.4)	3 (0.6)	0	2 (0.4)	193 (36.1)	169 (31.6)	106 (19.8)	44 (8.2)	14 (2.6)	7 (1.3)	2 (0.4)
Personal values	186 (34.8)	177 (33.1)	113 (21.1)	37 (6.9)	10 (1.9)	10 (1.9)	2 (0.4)	193 (36.1)	159 (29.7)	112 (20.9)	38 (7.1)	20 (3.7)	10 (1.9)	3 (0.6)
My experience	131 (24.5)	236 (44.1)	123 (23.0)	31 (5.8)	9 (1.7)	3 (0.6)	2 (0.4)	79 (14.8)	188 (35.1)	151 (28.2)	90 (16.8)	18 (3.4)	6 (1.1)	3 (0.6)
Evidence from studies and guidelines	115 (21.5)	194 (36.3)	149 (27.9)	57 (10.7)	14 (2.6)	4 (0.7)	2 (0.4)	81 (15.1)	165 (30.8)	144 (26.9)	101 (18.9)	30 (5.6)	12 (2.2)	2 (0.4)
Instruction by physician	105 (19.6)	233 (43.6)	149 (27.9)	35 (6.5)	9 (1.7)	2 (0.4)	3 (0.6)	101 (18.9)	172 (32.1)	152 (28.4)	74 (13.8)	27 (5.0)	6 (1.1)	3 (0.6)
Instruction by pharmacy manager	91 (17.0)	132 (24.7)	113 (21.1)	77 (14.4)	45 (8.4)	72 (13.5)	5 (0.9)	85 (15.9)	114 (21.3)	123 (23.0)	99 (18.5)	34 (6.4)	74 (13.8)	6 (1.1)
Patient’s wish	49 (9.2)	133 (24.9)	222 (41.5)	99 (18.5)	24 (4.5)	6 (1.1)	2 (0.4)	42 (7.9)	112 (20.9)	186 (34.8)	133 (24.9)	50 (9.3)	10 (1.9)	2 (0.4)
Personnel and time resources of the pharmacy	64 (12.0)	119 (22.2)	156 (29.2)	122 (22.8)	53 (9.9)	19 (3.6)	2 (0.4)	41 (7.7)	114 (21.3)	146 (27.3)	141 (26.4)	68 (12.7)	21 (3.9)	4 (0.7)
Solidarity principle	35 (6.5)	68 (12.7)	116 (21.7)	148 (27.7)	89 (16.6)	75 (14.0)	4 (0.7)	21 (3.9)	63 (11.8)	94 (17.6)	179 (33.5)	107 (20.0)	66 (12.3)	5 (0.9)
Commercial considerations	17 (3.2)	56 (10.5)	128 (23.9)	185 (34.6)	115 (21.5)	32 (6.0)	2 (0.4)	16 (3.0)	59 (11.0)	118 (22.1)	167 (31.2)	133 (24.9)	40 (7.5)	2 (0.4)
Religious beliefs	14 (2.6)	28 (5.2)	46 (8.6)	80 (15.0)	108 (20.2)	256 (47.6)	3 (0.6)	20 (3.7)	34 (6.4)	45 (8.4)	92 (17.2)	94 (17.6)	248 (46.4)	2 (0.4)

### Previous education in pharmacy ethics and a wish for further education in pharmacy ethics

A total of 341 out of the 535 participants (63.7%) stated that they had had no previous education in pharmacy ethics; 136 participants (25.4%) had had elements about pharmacy ethics during their studies, 30 (5.6%) in continuing professional education and 63 (11.8%) in personal education. Most of the participants (449, 83.9%) would prefer more education in pharmacy ethics (answered “yes” or “rather yes”), and 417 participants (77.9%) thought that this education could help them in their everyday professional life (answered “yes” or “rather yes”).

## Discussion

This survey represents one of the first structured cross-sectional studies of ethical conflicts in community pharmacy practice in Germany. It showed that pharmacists in community pharmacies are frequently affected by burdensome ethical conflicts in their daily patient care. The fear of negative consequences for the patient is especially perceived as burdensome. Hence, it is not surprising that personal values were rated similarly important in decision-making to objective considerations such as pharmaceutical knowledge and legal requirements.

### Frequency and burden of ethical conflicts

Ethical conflicts are a part of the daily work in the community pharmacy but vary depending on the structure and organization of the pharmacy landscape in the respective country [4, 14, 15, 18–20]. In this survey, the conflict “Generic drug is not most suitable” appeared to be the most frequent. This is not surprising, since there are approximately 39,500 health insurance-specific contracts concerning generic drugs in Germany [21]. The setting has the potential for more ethical conflicts than those investigated [3, 4]. Additionally, they are not always consciously perceived [12]. Thus, it can be assumed that ethical conflicts occur more frequently and in a greater variety than those investigated in this study.

Notably, pharmacists rated all of the predefined ethical conflicts to be burdensome. The fear of negative consequences for the patient was the predominant factor in the perceived burden. This seems logical as the survey aimed at investigating patient care situations and “patients’ best interest” is a core value of pharmacists [15, 22]. “Missing prescription for a needed drug” is the only conflict in which the main rated reason for the heavy burden is the fear of potential negative consequences for the pharmacist themself. In this situation, the fear of fines and imprisonment or even the loss of the licence to practice seems to outweigh the concerns for the patient. The 24-h availability of medical services in Germany enables patients to get a prescription and redeem it in a pharmacy at any time. This could explain why the personal consequences for the pharmacist are experienced more seriously.

The question arises whether there is a difference in terms of the burden whether prescription or OTC drugs are involved. The dispensing of OTC drugs in Germany is the sole domain of community pharmacists. Although the former are supposedly more harmless than prescription drugs, many problems, such as abuse and addiction all the way to hospitalization, arise precisely from this trivialization [23–25]. In our survey, the conflicts limited to OTC drugs were rated to occur frequently and are mainly associated with a rather weak up to rather heavy burden. In conclusion, dispensing OTC drugs is a potentially burdensome and frequent conflict field which is unique to the community pharmacy.

### Decision-making in ethical conflicts

Ethical decision-making is an important prerequisite for ethical behaviour [12]. The decision-making process per se is complex and depends on various factors, such as age, personal values, work experience and moral reasoning skills [26, 27]. It is known that pharmacists tend to base their decisions on their pharmaceutical knowledge and legal requirements [12, 22]. The survey confirmed these findings. Pharmaceutical knowledge thereby was considered to be more important than evidence from studies and guidelines. This seems to be a contradiction, as the former and latter items are related to each other. The rating might be influenced by pharmacy education. Similar to other healthcare professionals, pharmacists see themselves confronted with the challenge of implementing evidence-based practice in education [28, 29]. This is aggravated by the fact that scientific-based contents in Germany are still predominant in pharmacy education in contrast to clinical or medical lectures where evidence-based pharmacy is usually imparted [30].

Interestingly, in this survey the patient’s wish appears comparatively unimportant to the other items queried. It should be considered that the patient’s initial wish (e.g. for an OTC drug) might be influenced by advertising, recommendations from acquaintances or the patient’s own experience [31]. Pharmacists, therefore, have to medically and pharmaceutically examine the patient’s wish in a conversation. If the pharmacist concludes that an alternative would be better for the patient, this might be a frequent constellation in which the patient’s wish, which can be an expression of the patient’s autonomy, may be considered to be less important than pharmaceutical knowledge, which is thereby used with the aim of beneficence [32]. In contrast to this ethical approach, pharmacists find themselves frequently accused of commercial interests [33, 34]. Commercial considerations were chiefly rated as having a rather weak influence on decision-making in our survey and were in second last place in terms of all considerations queried.

### Implications for practice

The survey showed that the fear of negative consequences for the patient is already a burdensome factor in everyday ethical conflicts. Pharmacists will face increasingly severe ethical conflicts directly affecting the patient because the number of pharmaceutical services will increase within the next few years [35, 36]. This has the potential to cause or worsen pharmacists’ burden. Research performed with nurses has shown that this has the potential to have a negative impact on both the healthcare professional and patients [10, 37]. Consequently, there is a great need for preventive strategies which support pharmacists in decision-making. Typically, a code of ethics could be a decision-making aid, but there is no national code of pharmacy ethics in Germany [20]. Therefore, it is even more important to support decision-making through ethical education. Most of the participants stated that they had not received any ethics education but would like further information and consider it helpful in their daily life. This is surprising, because moral or ethical competence is frequently discussed in relation to the professionalism of healthcare professionals and also regarding pharmacists [38–41]. Concerning Germany, the regulation on the licensing of pharmacists schedules ethical aspects as a part of clinical pharmacy in university education and are, therefore compulsory [42]. The results regarding the previous education in pharmacy ethics shows that this content has not been lastingly conveyed or even was not conveyed to the pharmacists. The latter is possible as some of the participants could have finished their education before the introduction of clinical pharmacy in the licensing regulation. Lectures and practical exercises, such as ethical dilemma case discussions, have already been shown to have a positive impact on the ethical skills of pharmacy students [43–45]. A more widespread implementation, including continuing education, would be desirable.

### Limitations

Firstly, the questionnaire was not comprehensively validated. However, face and theoretical validity as well as usability were assessed through the literature, expert panel discussion, and cognitive and conventional pretesting. Secondly, the survey was voluntary. It can be assumed that the participating pharmacists were interested in the topic and showed a high awareness of ethical conflicts, which introduces non-response bias. Thirdly, the sample size was n = 535 and the distribution of participants across the country was very heterogeneous, limiting the generalizability of the results. Fourthly, as this survey primarily aimed at presenting the given ratings, no detailed evaluation in terms of an ethical theory was provided. Despite these limitations, this is the first survey on the topic in Germany, providing initial insights into this largely neglected issue.

## Conclusion

Pharmacists in community pharmacies are burdened by ethical conflicts in everyday patient care situations. Special issues arise from the unique field of OTC counselling. Concerns for their patients are burdensome factors. To prevent distress, a more widespread integration of pharmacy ethics in education would be desirable.

## Supplementary Information

Supplementary file 1
