# Supplementary material for: Ethical conflicts in patient care situations of community pharmacists: a cross-sectional online survey
Source: Int J Clin Pharm. 2024 Sep 6;46(6):1500–13. doi: 10.1007/s11096-024-01797-9 (PMC11576625; doi:10.1007/s11096-024-01797-9)
Supplement: Supplementary file 1 [file 11096_2024_1797_MOESM1_ESM.docx]

**Supplement 1**

Survey (own translation, original survey was performed in German.)

| **Page, Heading,** introductory explanation | **Content/ Items** | **Possible answers** |
| --- | --- | --- |
| **1 Survey on ethical conflicts in community pharmacies** | Dear pharmacists,  dear pharmacists in internship,  dear colleagues,  Difficult situations sometimes arise in patient care, for example, a patient urgently needs a medicine and does not have a prescription, the health insurance company does not reimburse the drug tolerated by the patient or there is a suspicion that a patient is misusing a drug. In these and similar situations, there are several options available, but the decision is difficult. You want to do the "right" thing – but what is right is not so easy to answer. This is what we call an ethical conflict. With this survey, we would like your opinion on such situations in your everyday care of patients. We are interested in your personal perception. There is no right or wrong answer. Please answer the questions spontaneously and as honestly as possible. We ask you to take part only once. The questionnaire takes about 20 minutes to complete. In the interests of better readability, the masculine form of personal nouns has mainly been chosen, but both genders are meant equally.  Thank you very much for your support,  Dipl.-Pharm. Kathrin Wernecke, Dr. Susanne Schiek and Prof. Dr. Thilo Bertsche  University of Leipzig, Faculty of Medicine, Institute of Pharmacy, Clinical Pharmacy, Brüderstr. 32, 04103 Leipzig  Note on data protection:  No personal data is collected. The IP address cannot be traced either. Therefore, it is not possible to draw conclusions about a specific person, and your answer remains anonymous at all times. The data will be analysed for purely scientific purposes by employees of the Clinical Pharmacy Department of the University of Leipzig. The data will not be passed on to third parties. Due to anonymity, it is not possible to delete individual data after participation. The survey was approved by the Medical Faculty of the University of Leipzig.  Please confirm:  I have read and understood the privacy policy. *[compulsory check mark]* | |
| **2 General view of ethical conflicts** | Please decide spontaneously: How often do you experience situations in your everyday patient care in which you personally find yourself in an ethical conflict*? Please select the option that is most likely to apply.  *Situations in which ethical principles conflict with other considerations (e.g. legal regulations,  commercial considerations). | Never  At least once a year  At least once a quarter  At least once a month  At least once a week  At least once a day |
|  | Please decide spontaneously: How burdensome do you normally perceive these situations? Please select the option that is most likely to apply. | No burden  Rather weak burden  Weak burden  Rather heavy burden  Heavy burden  Very heavy burden |
| **3–5 Frequency**  Some everyday situations are listed below | Please decide spontaneously for each situation: How often do you experience the situation mentioned in your everyday professional life? Please select the option that is most likely to apply.  *[5 of the 15 ethical conflicts from Table 2 were randomly shown per page 3–5]* | Never  At least once a year  At least once a quarter  At least once a month  At least once a week  At least once a day |
| **6–8 Burden**  The situations are now listed again below. | Please decide spontaneously for each situation: How much of a burden do you normally perceive in the situation mentioned? Please select the option that is most likely to apply.  *[5 of the 15 ethical conflicts from Table 2 were randomly shown per page 6–8]* | No burden  Rather weak burden  Weak burden  Rather heavy burden  Heavy burden  Very heavy burden |
| **9**  *Only available, for the situations that were rated with a (very) heavy burden by the participant* | You have indicated that you are perceiving a (very) heavy burden in the following situation:  *[ethical conflict rated with a (very) heavy burden 1]*  Please now tick why you perceive a (very) heavy burden in this situation [multiple choice possible]  This situation is very stressful because of the potential consequences for ...  --------------------------------------------------------  You have indicated that you are perceiving a (very) heavy burden in the following situation:  *[ethical conflict rated with a (very) heavy burden 2]*  Please now tick why you perceive a (very) heavy burden in this situation [multiple choice possible]  This situation is very stressful because of the potential consequences for ...  *[repeated depending on the number of conflicts rated with a (very) heavy burden by the participant]* | ... the patient.*  ... the pharmacy.*  ... myself.*  … the physician.**  … the health insurance.**  … a third person (child).**  None of the above options apply.*  **These options were always available.*  ***Only available if applicable to the respective conflict (see Table 4)* |
| **10 Considerations and guidelines in everyday life**  You have now seen various situations that can bring you into conflict.  Here are a few considerations that you can use for your decision in such situations. | How strong is the influence of these considerations on your decision [in an ethical conflict situation] in everyday life? Please select the option that is most likely to apply.  *[all 12 decision-making considerations were shown in a random order]*  *The item “solidary principle” was explained by this footnote:*  *is the principle of statutory health insurance, according to which a citizen does not have to pay for the costs themselves in the event of illness, but all members of the community do. This refers to the effects that an action has on this community of insured people. | No influence  Weak influence  Rather weak influence  Rather strong influence  Strong influence  Very strong influence |
| **11 Considerations and guidelines in everyday life**  Here are the considerations and guidelines that you can use for your decision listed again. | How much of a burden do you feel when you “have to” act against these considerations in everyday life? Please select the option that is most likely to apply.  *[all 12 decision-making considerations were shown in a random order]*  *The item “solidary principle” was explained by this footnote:*  *is the principle of statutory health insurance, according to which a citizen does not have to pay for the costs themselves in the event of illness, but all members of the community do. This refers to the effects that an action has on this community of insured people. | No burden  Weak burden  Rather weak burden  Rather heavy burden  Heavy burden  Very heavy burden |
| **12 Pharmacy ethics in training and practice** | Have you already had content on the topic of pharmacy ethics in:  [multiple answers possible] | Study  Continuing professional education (e.g. seminar organized by the chamber)  personal education (e.g. literature)  None of the above options apply. |
|  | Would you like more information on the topic of pharmacy ethics? | Yes  Rather yes  Rather no  No  I cannot answer this. |
|  | Do you think this knowledge could help you in your everyday work? | Yes  Rather yes  Rather no  No  I cannot answer this. |
| **13 Socio-demographic data**  In the following, we would like to ask for some more statistical data for the purpose of scientific evaluation. | How old are you? | … years *[free text]* |
|  | Which gender do you feel you belong to? | Male  Female  Diverse |
|  | In which function do you work in the community pharmacy? | Employed pharmacist  Owner of a pharmacy  Branch managing pharmacist  Pharmacist in internship  Other |
|  | In which federal state do you work? | *[Drop down menu with all 16 federal states of Germany]* |
|  | How big is the location where you work? | Metropolis (>100.000 citizens)  Town (>20.000 citizens)  Small town (>5.000 citizens)  Rural community (<5.000 citizens |
|  | How much professional experience do you already have in a community pharmacy?  (Please only include the years that you have actually worked in a community pharmacy. The internship is included. Please deduct parental leave(s) or employment in other pharmaceutical fields). | … years *[free text]* |
|  | Please estimate what proportion of your working time is spent caring for patients?  (This includes all activities to provide a patient directly with drugs or aids). | *[Drop down menu]* %  0–10  11–20  21–30  31–40  41–50  51–60  61–70  71–80  81–90  91–100 |
| **14** | Is there anything else you would like to tell us about ethical conflicts in public pharmacies? Then this is the place for it. | *[free text]* |
| **15** | Thank you for your participation!  Your answers have been saved. We would like to thank you very much for your help. You have helped us to gain an insight into the everyday ethical conflicts of a pharmacist.  We would like to thank Stephan Nadolny and Prof. Dr. med. Jan Schildmann (Institute for History and Ethics in Medicine, Martin Luther University Halle-Wittenberg) for their support in developing the survey.  Dipl.-Pharm. Kathrin Wernecke, Dr. Susanne Schiek and Prof. Dr. Thilo Bertsche  University of Leipzig, Faculty of Medicine, Institute of Pharmacy, Clinical Pharmacy, Brüderstr. 32, 04103 Leipzig  You can now close the browser window. | |

Adjustments after pretests:

Multiple choices of the parties involved were allowed on page 9 (instead of the option “several options apply”). The decision-making considerations (page 10 and 11) were modified as follows: “pharmaceutical knowledge” and “evidence” were separated; “organizational procedure and pharmacy routine” were changed to “personnel and time resources”; “patient’s wish or behaviour” was changed to “patient’s wish”; “impact on society and solidarity” was changed to “solidarity principle” and a definition of solidarity principle was added; and “my experience” and “religious values” were added.
